# A Compartmented Flow Microreactor System for Automated Optimization of Bioprocesses Applying Immobilized Enzymes

**DOI:** 10.3389/fbioe.2018.00189

**Published:** 2018-12-04

**Authors:** Raphael Heinzler, Jonas Hübner, Thomas Fischöder, Lothar Elling, Matthias Franzreb

**Affiliations:** ^1^Institute of Functional Interfaces, Karlsruhe Institute of Technology, Karlsruhe, Germany; ^2^Laboratory for Biomaterials, Institute for Biotechnology and Helmholtz Institute for Biomedical Engineering, RWTH Aachen University, Aachen, Germany

**Keywords:** bioreactor, magnetic particles, process development and optimization, immobilized enzymatic reactor, automated microreaction system

## Abstract

In the course of their development, industrial biocatalysis processes have to be optimized in small-scale, e. g., within microfluidic bioreactors. Recently, we introduced a novel microfluidic reactor device, which can handle defined reaction compartments of up to 250 μL in combination with magnetic micro carriers. By transferring the magnetic carriers between subsequent compartments of differing compositions, small scale synthesis, and bioanalytical assays can be conducted. In the current work, this device is modified and extended to broaden its application range to the screening and optimization of bioprocesses applying immobilized enzymes. Besides scaling the maximum compartment volume up to 3 mL, a temperature control module, as well as a focused infrared spot were integrated. By adjusting the pump rate, compartment volumes can be accurately dosed with an error <5% and adjusted to the requested temperature within less than a minute. For demonstration of bioprocess parameter optimization within such compartments, the influence of pH, temperature, substrate concentration, and enzyme carrier loading was automatically screened for the case of transferring UDP-Gal onto N-acetylglucosamine linked to a tert-butyloxycarbonyl protected amino group using immobilized β1,4-galactosyltransferase-1. In addition, multiple recycling of the enzyme carriers and the use of increased compartment volumes also allows the simple production of preparative amounts of reaction products.

## Introduction

Microfluidic reaction devices are widely used for analysis, synthesis, diagnostics, biosensing, and a broad range of other applications in the pharmaceutical and healthcare industries. They enable the miniaturization, integration, and automation of biochemical assays with real time or end-of-line analytical measurements (Haeberle and Zengerle, 2007; Zhang and Haswell, 2007; Malic et al., 2010). Considerable attention was attracted by immobilized microfluidic enzymatic reactors (IMER) which have several benefits like reduced process costs, increased reaction speed, and improved control of process parameters, due to the their small volumes (Krenková and Foret, 2004; Matosevic et al., 2011). In addition immobilized enzymes often show improved storage stability and simplified reuse, helping the IMER concept to become more and more interesting for biotechnological applications (Miyazaki and Maeda, 2006; Urban et al., 2006; Jussen et al., 2016). By using commonly used immobilization techniques such as the complexing of genetically engineered polyhistidine tags of the enzyme to functional groups on the surface of a carrier, immobilized enzymes can show activities close to the ones of free enzymes in solution due to the highly specific orientation provided by the His_6_ linkage (Cha et al., 2005). As carriers, magnetic particles are well-suited because of their easy separation by magnetic fields, thus resulting in an enzyme free product without time-consuming and expensive purification steps. Magnetic particles with immobilized enzyme are already used in analytical biotechnology as biochemical detectors for sugar (Nomura et al., 2004) and protein (Choi et al., 2002). In a previous article, we introduced a novel microfluidic reactor device allowing contactless separation and resuspension of magnetic particles in combination with segmented microfluidics (Hübner et al., 2015, 2017). By the use of automated syringe pumps and multiport valves the device creates aqueous compartments in disposable tubings as reaction compartments with volumes around 10 to 250 μL. The magnetic particles placed in these compartments are controlled by permanent- and electromagnetic fields.

In this work, technical upgrades of the reactor device are presented allowing to work with volumes up to 3 mL and to control the temperature within the reaction zone. By adding an automated temperature controlled unit, it is now possible to analyze and optimize reaction conditions of enzymes immobilized on magnetic particles fully automated. As a proof of concept, the catalytic activity of immobilized β-1,4-galactosyltransferase (β4GalT) was tested at different temperatures, pH values, and loadings.

## Materials and Methods

### Original Reactor Device

**Figure 1:**
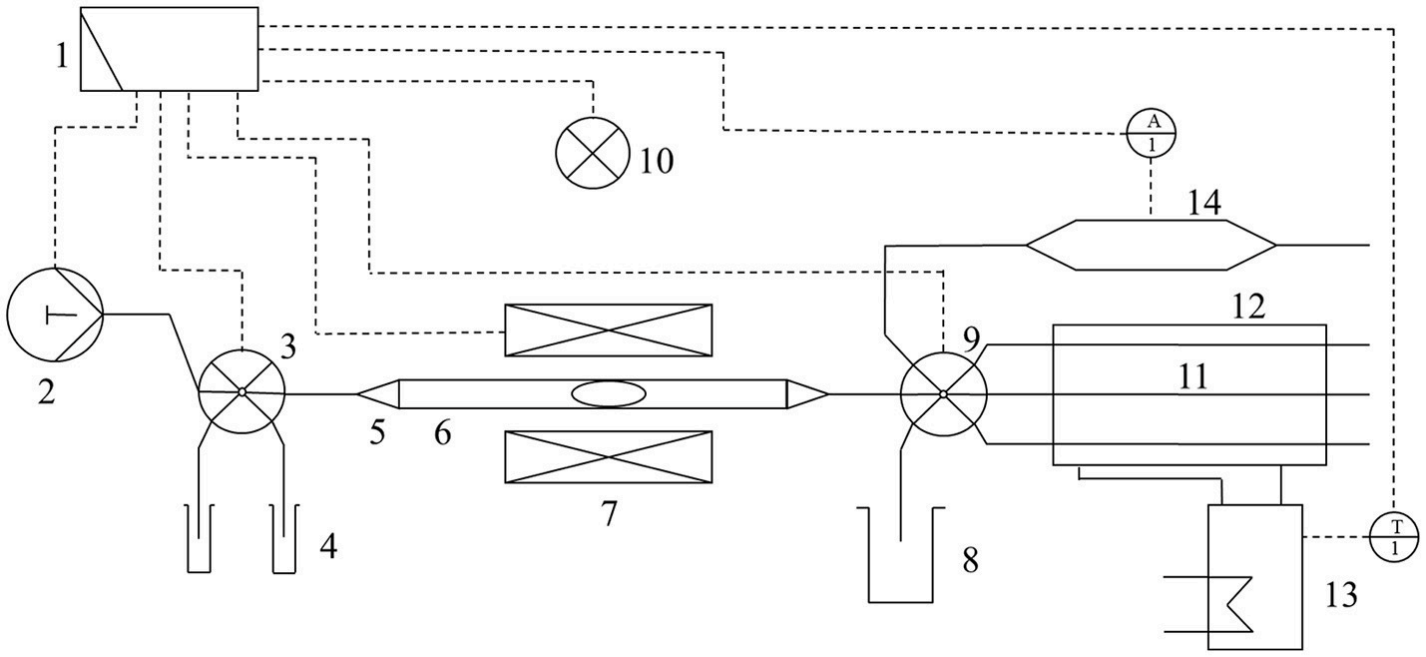
Setup of the rector device as a general flow scheme. All modules of the device are connected to a PC (1). Syringe pumps (2) connected to a multiport valve (3) transport all process relevant fluids and magnetic enzyme immobilisates (MEI) from reservoirs (4) via tube adapters reducing compression unions (5) to the tube (6) passing the magnetic field module (MFM) (7). Waste can be removed to containers (8) via another multiport valve (9). Temperature-dependent short term reactions can be performed directly inside the MFM through heating by an infrared spot (10). Alternatively, for temperature-dependent long term reactions, the MEI can be transferred to several tubes (11) of the temperature control module (12), which is connected to a thermostat (13). The supernatant of a reaction can be transferred to a spectrometer module (14) for online analytics.

The principal of the microfluidic reactor device is based on creating well-defined aqueous reaction compartments by alternately pumping organic solvent and aqueous reaction solution within a tubing. Magnetic beads can be inserted in reaction compartments e.g., for purification or synthesis purposes. The reactor system (Figure 1) comprises several fluid system modules obtained from Cetoni (Korbußen, Germany). It consists of syringe pumps (10 and 50 mL), two valves (11 connections for 1/8" tubing), an analog and digital input/output (I/O) module and a spectrometer module for online analysis. All modules are controlled by the QmixElements automation software and are interconnected by fluorinated ethylene propylene (FEP) tubing with an outer diameter of 1/8" purchased from CS-Chromatographie Service (Langerwehe, Germany). Inside the magnetic field module (MFM), one of the tubes is guided between two electromagnetic coils, which are mounted on a 3D printed holder and connected to the I/O module, to be controlled by the software. A 3D printed lever is installed below the tube. The lever is moved by a servo and a permanent magnet array is mounted at the end of the lever. By moving the magnet array to the tube, magnetic beads used in a reaction compartment can be fixed and, by that, separated from surrounding fluid. Further details of the reactor device have been previously described (Hübner et al., 2017).

### Magnetic Microcarriers

PureCube Ni-IDA MagBeads were purchased from Cube Biotech GmbH (Monheim am Rhein, Germany) and used as enzyme carriers without further modification (Table 1).

**Table 1:** Magnet bead information according to manufacturer.

**Material**	**Magnetite (Fe_3_O_4_) beads coated with 6% cross linked agarose**
Affinity ligand	Ni-IDA
Magnetization	ferrimagnetic
Bead diameter	25–30 μm
Binding capacity	up to 70 mg (IDA) His-tagged protein/mL of settled beads

### Enzymes and Chemicals

The enzyme studied in this work is a fusion construct of an *N*-terminal polyhistidine (His_6_)-tag, a lipase pre-propeptide from *Staphylococcus hyicus* and the catalytic domain of the human β1,4-galactosyltransferase-1 (β4GalT). The enzyme expression of recombinant human β4GalT (EC 2.4.1.38) in *Escherichia coli* was done by the project partner Prof. LE, Helmholtz-Institute for Biomedical Engineering, RWTH Aachen University, Germany, as described by Sauerzapfe et al. (2008) and Fischöder et al. (2017). Also the linker-modified substrate *N*-acetylglucosamine with a *tert*-butyloxycarbonyl protected amino group (GlcNAc-linker-*t*Boc) was supplied from Prof. Vladimir Kren (Institute of Microbiology, Czech Academy of Sciences) and produced as described by Sauerzapfe et al. (2009). Inhibitory byproducts were removed by Alkaline Phosphatase (FastAP) purchased from Thermo Fisher Scientific (Rockford, USA). All chemicals were analytical grade and used without further purification. The water used for all experiments was deionized and purified using a Milli-Q Ultrapure system from Merck Millipore KGaA (Darmstadt, Germany).

### Methods

#### Immobilization of β4GalT Enzyme

The His-tagged β4GalT was immobilized onto the IDA-Ni functionalized surfaces of the magnetic particles. For the immobilization, 5 μL particle slurry were used to bind 25/50/75/150 μg β4GalT enzyme resulting in a theoretical loading of 5/10/15/30 g β4GalT per L particle slurry. The immobilization reaction was performed in 1.5 mL Eppendorf tubes (Wesseling-Berzdorf, Germany) and done in duplicates. After the immobilization step, the samples were washed one time with (0.5 mL) 1 M NaCl buffer and two times with storage/reaction buffer (0.5 mL) 100/25 mM HEPES/KCl buffer pH 7.5 to desorb non-covalently adsorbed enzymes. The enzyme-particles were diluted to 10 mL particle slurry per L storage buffer and stored at 4°C. To calculate the amount of bound enzyme, the protein content in the incubation- and washing-supernatants was determined using the bicinchoninic acid (BCA) Protein Assay Reagent Kit from Thermo Scientific Pierce (Rockford, USA) according to manufacturer's instructions.

#### Activity Assay of Free β4GalT

Tests to verify the activity of the free β4GalT qualitatively and to compare their activity quantitatively to the immobilized ones used in the microreactor system, were performed in 1.5 mL Eppendorf tubes according to Rech et al. (2011) and Fischöder et al. (2017). Components of the reaction mixture, except the enzyme solution, were prepared and pre-incubated for 5 min at 30°C in a volume of 30 μL. The reaction was started by the addition of 20 μL enzyme solution (0.05 g/L) pre-heated to 30°C for 5 min in deionized water, reaching the final volume of 50 μL. The final concentrations in the standard reaction at 30°C were: 100/25 mM HEPES/KCl pH 7.5, 6.52 mM MnCl_2_, 6.52 mM UDP-Gal, 5 mM GlcNAc-linker-*t*Boc, and 1 U Fast-AP. Components in the reaction mixture were varied according to the investigated parameters. The reaction was carried out in a temperature controlled shaker (Eppendorf® Thermomixer) at 1,400 rpm. To stop the reaction, samples of 6 μL were taken from the reaction mixture at defined time points and heated at 95°C in preheated reaction vessels in another temperature-controlled shaker at 500 rpm for 5 min. Denaturated proteins were removed by centrifugation at 4°C and 10,000 rpm for 5 min. Supernatant with a volume of 2.5 μL was transferred to a HPLC vial containing 122.5 μL of a mixture of acetonitrile (ACN) and Milli-Q with 0.1 % formic acid (MFA) in a ration 26/74 ACN/MFA. The injection in a HPLC system (Agilent 1,100 series, Waldbronn, Germany) was done with 30 μL of sample, which was measured at 254 nm at a flow rate of 0.3 mL/min. The concentration of the substrate GlcNAc-linker-*t*Boc and the product Galβ1-4GlcNAc(LacNAc)-linker-*t*Boc were calculated by the ratio of each peak area compared to the sum of the two peak areas and the corresponding calibration curve of standards. From the resultant concentrations, the activity was calculated by the increase of LacNAc-linker-*t*Boc over time. One enzyme unit correlates with 1 μmol product per minute. The mass specific activity U/mg was calculated for one enzyme unit per mg immobilized enzyme.

#### Activity Assay of Immobilized β4GalT

To characterize the activity of the β4GalT -particles in the microreactor system with regard to pH, temperature, and loading, magnetic enzyme immobilisates (MEI) were pumped into the reaction tubing of the system. The MEI were separated from the storage buffer (as described before; Hübner et al., 2017), resuspended in 100 μL of a preheated reaction buffer compartment and pumped to the temperature control module (TCM). Details of the IR-unit for preheating and the TCM are discussed in section describing the modified reactor system. For each test, 15 μg of immobilized enzyme were used in a 100 μL compartment. To avoid possible effects of enzyme deactivation, fresh MEI of the same batch were used in each test, except the tests on particle recycling. To test different temperatures, the activity of 15 g/L MEI was investigated at pH 7.5 and 25/30/35/40°C. The temperature stability of the MEI was verified by adding 5 mM GlcNAc-linker-*t*Boc and 5 mM UDP-Gal once again to the reaction solution of each temperature investigation after 30 min. Afterwards the product formation was observed for another 20 min to measure the remaining specific activity. Analyzes of the influence of different pH values were carried out at 30°C at pH 6.5/7.5/8.5. Reaction buffers with the respective pH values were connected to the inlet multiport valve and pumped into the system when needed. To test different enzyme loadings, MEI with a loading of 5/10/15/30 g/L were analyzed at pH 7.5 and 30°C.

For analysis of the reaction products, an offline as well as an online method were implemented. In case of the offline method samples of 10 μL of the reaction mixture were taken at defined time points, the MEI were separated by a magnet in a MagRack (GE Healthcare, GBR) and the reaction was stopped by transferring the supernatant into new reaction vessels heated to 95°C in a temperature controlled shaker at 500 rpm for 5 min. The following pretreatment and the analysis with the HPLC system was carried out as described above for the free enzymes. In case of the online method, photometric analysis was done in the reaction system. The analysis is in accordance to an assay described by Laaf et al. (2017) with minor changes due to the use of MEI. The formation of LacNAc-linker-*t*Boc was analyzed, using the pyruvate kinase/lactate dehydrogenase system for the detection of UDP, which is produced equimolar to LacNAc-linker-*t*Boc. Therefore, the decrease of the NADH+H^+^ concentration is equimolar to the increase of the LacNAc-linker-*t*Boc concentration. A 100 μL reaction compartment contained 0.1 M ammonium acetate/HEPES (pH 7.4), 25 mM KCl, 2 mM K2PO4, 4 mM MgCl2, 2 mM MnCl2, 5 mM GlcNAc-linker-*t*Boc, 5 mM UDP-Gal, 3 mM phosphoenol pyruvate, 1.5 mM NADH+H^+^, 5U pyruvate kinase, 5 U lactate dehydrogenase. For the online analysis in the reactor, the 100 μL reaction compartment is pumped back from the TCM into the MFM and the MEI were separated, followed by pumping the reaction compartment into the integrated UV/VIS module for measurement. Afterwards the reaction compartment is transferred back to the MFM into a position where it covers the separated MEI. Finally, the MEI are resuspended in the reaction compartment and the suspension is transferred back into the TCM. The whole analysis step takes around 60 s after which the enzymatic reaction can continue without particle loss or reduction of the compartment volume.

## Results and Discussion

### Modification and Extension of the Microfluidic Reaction Device

The original reactor device was designed for analytical purposes using small sample and reaction compartment volumes of 10–250 μL. In contrast, the current work and reactor design is focused on automated conduction of different tasks of enzymatic bioprocess development using immobilized biocatalysts. Among these tasks are the screening for optimum process parameters, the determination of process kinetics as well as investigations regarding catalyst reuse and small scale multicycle product synthesis. Especially with regard to the last task mentioned, the device was modified to handle larger volumes and to allow a flexible temperature control of the reaction compartments.

The ability to scale the reaction compartments 20–50 times is realized by a replacement of the multiport heads of the valves as well as the tubing used as reaction channel. FEP tubes with an outer diameter of 1/4"−1/2" allow to create reaction compartments up to 3 mL. Accordingly, the size of the permanent magnet array and the tube holders had to be adjusted. The reaction tubes were connected to the valves by a self-constructed 3D printed adapter and 1/8" outer diameter tubes.

Temperature control during short term reactions conducted inside the MFM module is achieved by an infrared (IR) light (Optron GmbH, Garbsen, Germany) centered above the MFM. Additionally, next to the IR spot, an installed digital USB camera microscope allowed online process control within the reaction compartment. The IR light beams a regional spot of IR light on the reaction channel of the MFM in the size of a reaction compartment. In case of long-term enzymatic conversion reactions, a separate temperature control module (TCM) is connected to the reaction device. The TCM is a 70 mm long plastic tube with a 40 mm outer diameter and 30 mm inner diameter. The two covers at each side have one opening for water in- and outlet and up to four openings for FEP tubes with a 1/4" outer diameter. The FEP tubes are connected to the outlet multiport valve of the reaction system. The water in- and outlets are connected to a thermostat (PT31 Peltier Thermostat, A.KRÜSS Optronic GmbH, Hamburg, Germany), which is connected to the I/O module of the microreactor system via a Genuino MICRO single-board microcontroller (μC), purchased from Arduino (Turin, Italy), for the automated script-based process control. The plastic tube is held by two tailor-made 3D printed brackets, which are screwed to a PVC block. A servo, which is connected to the I/O module via a second μC, is attached to the PVC block and moves a 3D printed lever which is equipped with a strong permanent magnet. By approaching the lever including the magnet repeatedly to the top of the TCM, magnetic enzyme immobilisates (MEI) placed in tempered reaction compartments within the FEPs are suspended and well-mixed.

### Accuracy and Reproducibility of Aqueous Compartment Generation

**Figure 2:**
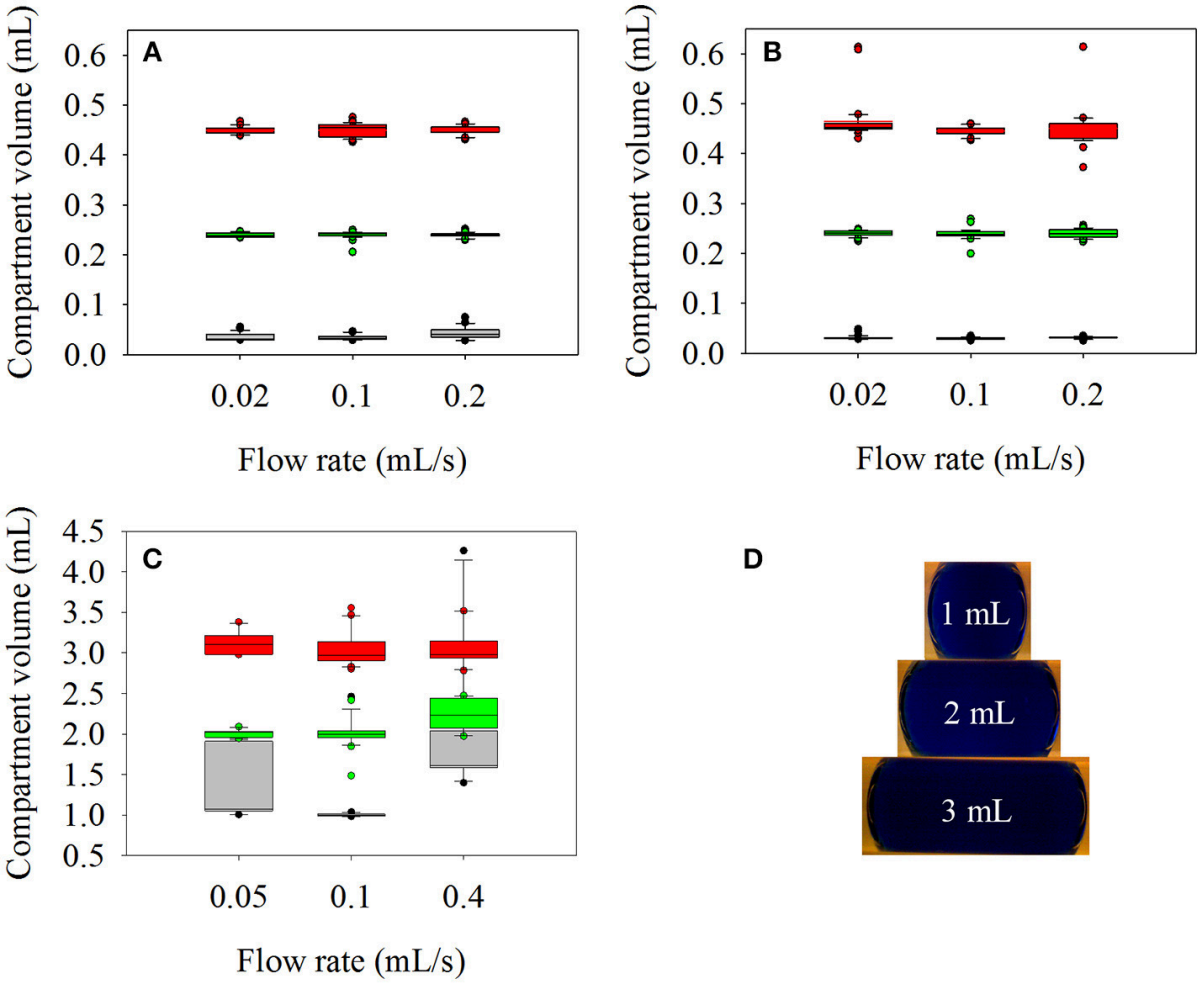
Accuracy and reproducibility of reaction compartment generation in dependence of applied separation fluid, tube diameter, flow rate, and compartment volume. Upper plots: measured volume of aqueous compartments adjusted to 0.03 mL (gray), 0.24 mL (green), and 0.45 mL (red) in a 1/4" OD tube separated by **(A)** ethyl acetate, and **(B)** anisole at flow rates ranging from 0.02 up to 0.2 mL/s Bottom plots: **(C)** Measured volume of aqueous compartments adjusted to 1 mL (gray), 2mL (green), and 3 mL (red) in a 1/2" OD tube separated by anisole at flow rates ranging from 0.05 up to 0.4 mL/s. **(D)** Pictures of 1, 2, and 3 mL aqueous compartments in a 1/2" OD tube separated by anisole.

The dosing accuracy of the original reaction device with 1/8” outer diameter (OD) tubes as reaction channel has been previously shown (Hübner et al., 2017). In this work, the reaction tubes were scaled up to 1/4 and 1/2" OD and could generate aqueous compartments up to 0.45 and 3 mL respectively (Figure 2). Based on a previous experience, ethyl acetate (EtOAc) was tested as separation fluid for the aqueous reaction compartments for the new tube sizes. While EtOAc resulted in accurate compartments in case of 1/4" tubes, it turned out to be impossible to generate defined aqueous compartments in the tubes with 1/2" outer and 0.47" (12 mm) inner diameter with EtOAc, n-decane, cyclohexane, polydimethylsiloxane oil or air as separation medium. Despite pumping the organic separator segments and aqueous reaction compartments consecutively in the tube, they quickly created horizontally separated layers over the whole tube length. Thus, anisole was chosen because its density is close to the density of water. Using anisole as separation fluid, it was possible to generate stable and defined reaction compartments in the 1/4" and the 1/2" OD tubes. To analyze reproducibility and dosing accuracy of the aqueous compartments within the 1/4" OD tube, 30 compartments of 0.03, 0.24, and 0.45 mL volume at flow rates of 0.02, 0.1, and 0.2 mL/s were generated in succession, respectively. The volume of the separator segments was 0.5 mL. For the tests in the 1/2" OD tube, 10 compartments of 1, 2, and 3 mL volume at flow rates of 0.05, 0.1, and 0.4 mL/s were generated with 2 mL separation compartments. Analysis was done as described previously (Hübner et al., 2017) using the digital USB camera microscope and image processing of the recorded video.

In the 1/4" OD tube, the standard deviations of the compartment volumes with EtOAc as separation fluid (Figure 2A) were <4% for 0.45 and 0.24 mL at all flow rates. If the pursued compartment volume is further decreased to 0.03 mL the standard deviation increases to 16% at 0.1 mL/s and 28% at 0.2 mL/s showing that the generation of compartment volumes below 0.1 mL should be avoided if larger tube diameters and high flow rates are used. In case of compartments separated with anisole (Figure 2B), standard deviations were <10%, except for 0.45 mL (18%) and 0.03 mL (14%) at the lowest flow rate of 0.02 mL/s.

For the compartment volumes separated with anisole in a 1/2" OD tube (Figure 2C), the standard deviations were below 10% for 2 and 3 mL compartments at all flow rates. Generating 1 mL compartments resulted in 30 and 46% standard deviation for 0.05 and 0.4 mL/s. At 0.1 mL/s it was possible to generate aqueous compartments with a volume standard deviation of only 1.5% except one time when two compartments merged. To prevent merging of small compartments, a higher volume of organic solvent between the aqueous compartments should be used. In summary, the results show good accuracy and volume reproducibility of compartments between 0.1 and 3 mL if appropriate flow rates are used during their generation.

### Characterization of Temperature Control Modules

**Figure 3:**
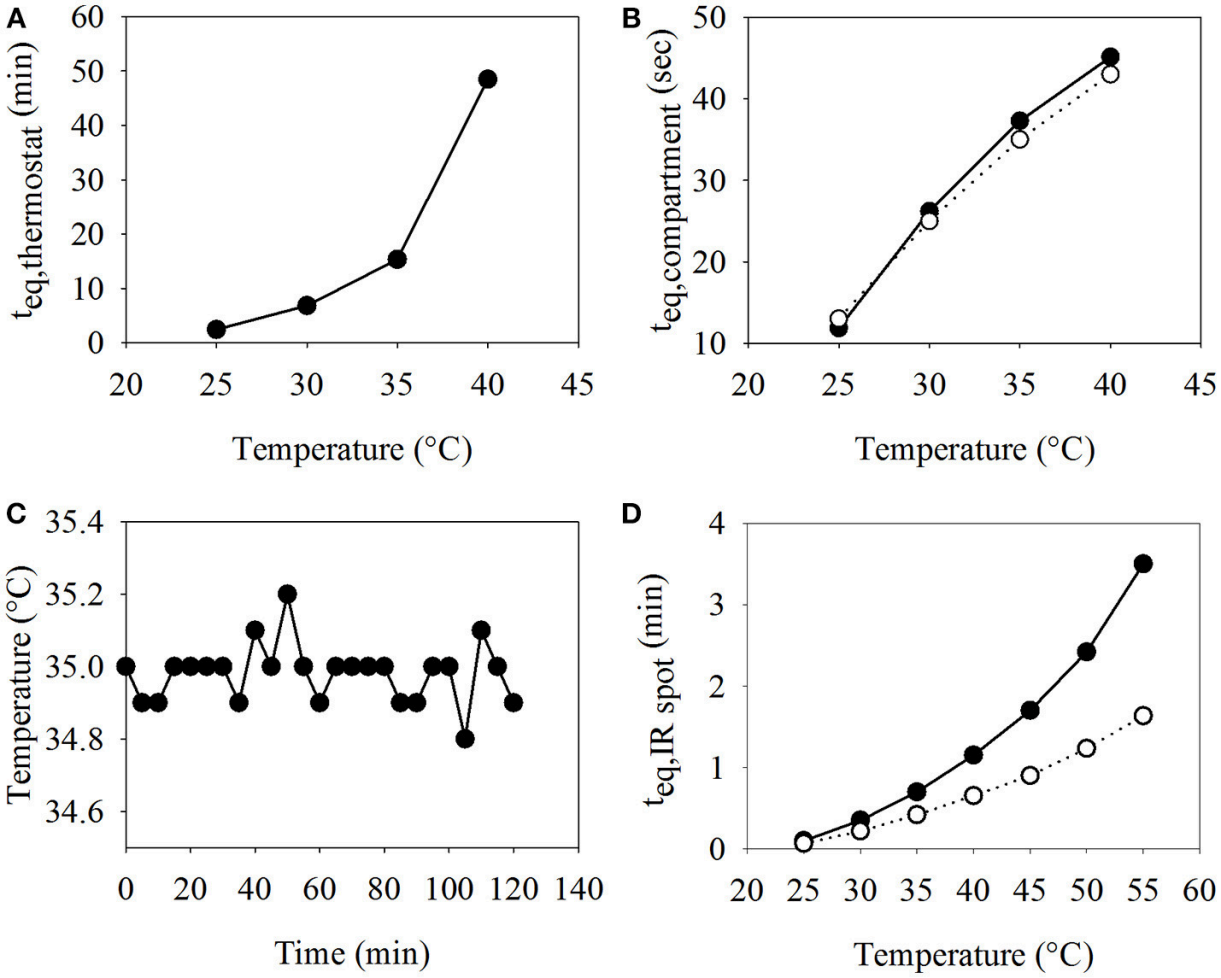
Performance curves for the TCM and IR light. **(A)** Plot of the time t_eq, thermostat_ the TCM needs to adjust to a given temperature when starting at room temperature, and **(B)** plot of the time t_eq, compartment_ it takes for an aqueous compartment to equilibrate with the temperature of the TCM—experimental measurement (-◦-) and theoretical calculation (-•-).**(C)** Thermal stability of the TCM over a period of 2 h at 35°C. **(D)** Plot of the time t_eq, IRspot_ an aqueous compartment irradiated by the IR light needs to adjust to the desired temperature at 30% (-•-) and 35% (-◦-) of maximum IR light power.

In order to run biocatalytic reactions at defined conditions or to screen optimal reaction temperatures, automated adjustment and stable control of the temperature of the reaction compartments plays a crucial role. With the developed temperature control module (TCM), the temperature within the cylindrical water bath housing up to four tubes with inset reaction compartments can be automatically set and maintained in a range of 8–40°C. TCM efficiency can be depicted by two performance curves: first the time the water bath requires to reach a certain temperature (up to a difference of 1°C) when starting at room temperature (22°C) (Figure 3A), and second, the time an aqueous compartment requires to equilibrate with the water bath temperature after being pumped into one of the holding tubes (Figure 3B). Another way to heat up a compartment is with IR light in the MFM. The temperature is controlled by setting the IR light power in percentage and can be adjusted to temperatures much higher than 40°C (Figure 3D).

Figure 3B shows that the time a compartment needs to equilibrate its temperature with the temperature in the TCM is small, with 25 s (26 s calculated) and 40 s to reach 30 and 40°C (45 s calculated), respectively. These values fit well to the theoretically calculated duration for temperature equilibration. The time it takes for the thermostat to heat up can be neglected, if it is started ~1 h before the start of the experiment. If the desired temperature is reached, the TCM can maintain it with high accuracy during the complete experimental run (see Figure 3C). The IR spot needs a certain amount of power to reach higher temperatures in acceptable time periods. For example, a reaction compartment placed within the IR spot could reach a temperature of 50°C within <3 min, when the power was adjusted to at least 30% of the maximum power, while it took more than 20 min in the case of a power adjustment to 25% (data not shown). On the other hand, using settings higher than 35% of the maximum power resulted in very fast temperature changes which are difficult to control and comprise the danger of overheating.

### Screening of Optimal Process Parameters

In order to demonstrate the suitability of the developed microfluidic device for screening optimal reaction conditions of enzymatic reactions, the pH, and temperature optimum of β4GalT was screened. Furthermore, the effect of different concentrations of the substrate UDP-Gal and the influence of the enzyme loading of the catalyst particles were investigated for the transfer reaction of immobilized β4GalT using UDP-Gal onto GlcNAc-linker-*t*Boc. For conducting the test series, it was only necessary to provide reaction solutions with different pH values or UDP-Gal concentrations, or MEI with different enzyme loadings. The following activity assays could be performed fully automated by the system. For comparison, free β4GalT was manually assayed under the same conditions in 1.5 mL Eppendorf tubes and a temperature-controlled shaker.

The range of operation parameters tested was chosen according to the optimal conditions of free β4GalT reported in literature (Lange et al., 2016). The effect of pH, temperature, and substrate concentration on the activity of free and immobilized β4GalT was examined in the pH range of 6.5–8.5 at 30°C, in the temperature range 25–40°C at pH 7.5, and with UDP-Gal concentrations of 0.25–6.52 mM at pH 7.5 and 30°C, all conducted applying MEI with 15 g/L enzyme loading. In addition the effect of the MEI enzyme loading was tested in the range of 5–30 g/L at pH 7.5, 30°C with a solution of 6.52 mM UDP-Gal and 5 mM GlcNAc-linker-*t*Boc as substrates in 100/25 mM HEPES/KCl buffer.

**Figure 4:**
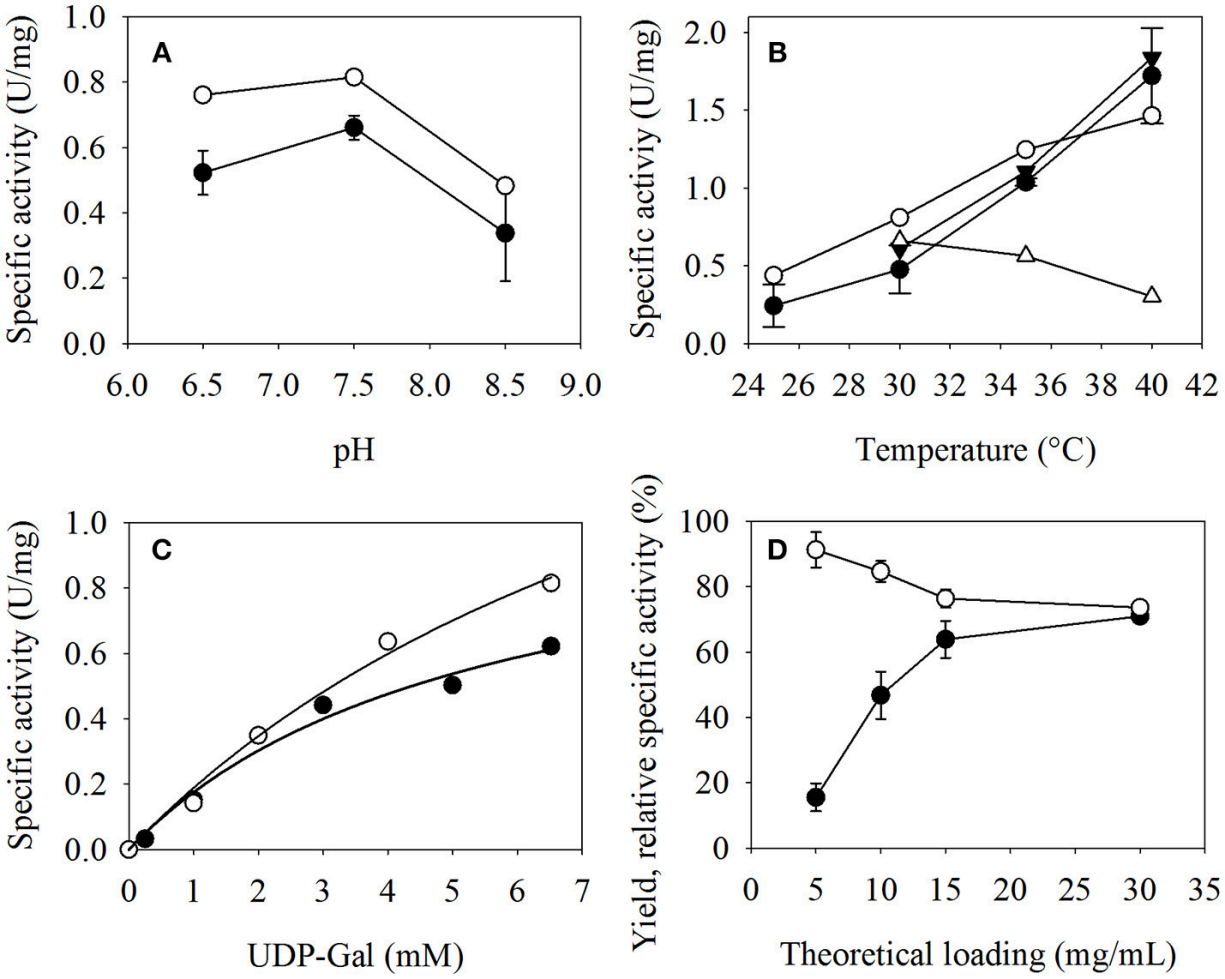
Specific activity of β4GalT at **(A)** different pH values with immobilized (-•-) and free (-◦-) enzymes, **(B)** different temperatures with immobilized (-•-) and (-◦-) free enzymes, and with GlcNAc-linker-*t*Boc addition after 30 min for immobilized (-▴-) and free (-Δ-) β4GalT, and **(C)** different concentrations of the substrate UDP-Gal, using immobilized (-•-) and free (-◦-) enzyme respectively. **(D)** Effect of the enzyme loading (mg enzyme per ml particle slurry) on the specific activity (-•-), and the binding (-◦-) of immobilized β4GalT. The activity assays were performed with **(A)** 15 g/L MEI enzyme loading, 100/25 mM HEPES/KCl (pH 6.5/7.5/8.5), 6.52 mM MnCl_2_, 6.52 mM UDP-Gal, and 5 mM GlcNAc-linker-*t*Boc as substrates and 1 U Fast-AP at 30°C; **(B)** 15 g/L MEI enzyme loading, 100/25 mM HEPES/KCl (pH 7.5), 6.52 mM MnCl_2_, 6.52 mM UDP-Gal, and 5 mM GlcNAc-linker-*t*Boc as substrates and 1 U Fast-AP at 25°C-40°C; **(C)** 15 g/L MEI enzyme loading, 100/25 mM HEPES/KCl (pH 7.5), 6.52 mM MnCl_2_, 0.25/1-5/6.52 mM UDP-Gal, and 5 mM GlcNAc-linker-*t*Boc as substrates and 1 U Fast-AP at 30°C; **(D)** 5/10/15/30 g/L MEI enzyme loading, 100/25 mM HEPES/KCl (pH 7.5), 6.52 mM MnCl_2_, 6.52 mM UDP-Gal, and 5 mM GlcNAc-linker-*t*Boc as substrates and 1 U Fast-AP at 30°C. For the assays at different temperatures, samples were taken for 30 min. At the 30 min mark, 6.52 mM UDP-Gal and 5 mM GlcNAc-linker-*t*Boc were added and samples were taken for another 20 min.

No difference between free and immobilized β4GalT could be detected regarding the pH value at which the enzyme showed the highest catalytic activity (Figure 4A). Therefore, the immobilization does not seem to alter the specific pH optimum of pH 7.5 of β4GalT. However, the immobilized enzymes showed an exponential increase of specific activity with increasing temperature from 25°C (0.39 U per mg immobilized enzyme) to 40°C (1.85 U per mg) (Figure 4B). In case of free β4GalT, the activity also increased from 25°C (1.11 U per mg free enzyme) to 40°C (2.93 U per mg), but with a decreasing slope. To determine, whether the enzymes are stable at this temperatures, additional substrate was added to the reaction after 30 min and the reaction was continued for another 20 min. The immobilized β4GalT displayed the same specific activity as in the first 30 min. In contrast, the specific activity of the free enzymes measured after 30 min reaction was already around 25% lower at 30°C (1.54 U per mg) and in case of 40°C the activity decreased even almost 80% (0.6 U per mg). This clearly shows the increased temperature stability and the shifted temperature optimum of the immobilized enzyme. The results are in accordance with e.g., the results of Zhou et al., who showed that his-tagged immobilized enzymes can gain more temperature resistance and lower temperature sensitivity (Zhou et al., 2017). They used his-tagged β-glucosidase immobilized on Fe_3_O_4_/PMG core/shell magnetic nanoparticles and examined their temperature stability in a range from 20–90°C (Prieto et al., 2015).

The assays with different UDP-Gal concentrations (Figure 4C) illustrate the around two times lower maximum reaction rate, which correlates with the specific activity, of the immobilized β4GalT (v_max_ = 1.12 U/mg) compared to the free enzyme (v_max_ = 2.19 U/mg) at 30°C. However, the Michaelis constant of the immobilized enzymes (K_m_ = 5.38 mM) is about half the value of the free (K_m_ = 10.62 mM). Kinetic constants were calculated by Sigma Plot 11 software (Systat Software GmbH, Erkrath, Germany) fitting the Michaelis-Menten equation v = v_max_^*^[S]/(K_m_ + [S]).

Different loadings of β4GalT were investigated, in order to study the effect of the density of immobilized enzymes on the particle surface (Figure 4D). For the lowest loading of 5 g β4GalT per L particle slurry, the activity yield after immobilization remains comparatively small at 16%. After doubling the amount of immobilized enzyme, the resulting activity was enhanced more than 3-fold, resulting in an activity yield of 54%. A further increase of the theoretical enzyme loading to 15 g/L and finally 30 g/L further enhanced the activity yield of the immobilization up to 70%. Regarding the relationship between enzyme loading, activity and binding yield, 15 mg β4GalT per mL particle slurry was chosen as standard condition for further experiments, because it combines a nearly optimal activity yield with low enzyme loss during the binding step. The decrease of the binding yield could indicate that there is a maximum of binding sites, which can be occupied. The enzymes, which could not bind or were only physically adsorbed, were lost in the following washing steps.

After characterizing the influence of the main process parameters onto the investigated biocatalytic reaction in a “one-factor-at-a-time” fashion, we extended the data basis by additional experiments and analyzed the system for its global optimum in the given parameter range by help of the statistics software MODDE 12.1 (Sartorius AG, Göttingen, Germany).

Applying the response surface methodology (RSM) the following equation, describing the variation of the specific acitivity in dependence of the parameters temperature, pH, enzyme loading of the magnetic particles and substrate concentration of UDP-Gal could be derived with a coefficient of determination *R*^2^ of 0.993.

$$\begin{array}{l} specific\;activity=\;-0.353\cdot T+0.00687\cdot T^{2}+1.168\cdot pH\\ -0.0889\cdot pH^{2}+\;0.0422\cdot loading\\ +0.179\cdot c_{UDP-Gal}-0.0138\cdot c_{UDP-Gal}^{2} \end{array}$$

**Figure 5:**
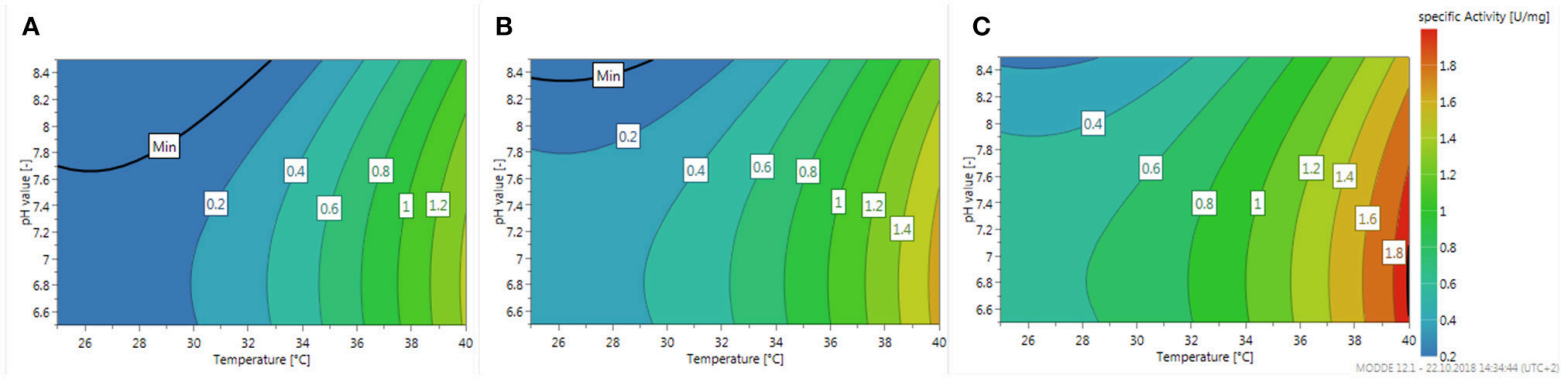
Contour plot of the specific activity of immobilized β4GalT for the case of transferring UDP-Gal onto N-acetylglucosamine linked to a tert-butyloxycarbonyl protected amino group. The specific activity marked as color is plotted against the pH value and the temperature at a loading of **(A)** 5, **(B)** 10, **(C)** 15 g enzyme per L particle slurry and a constant UDP-Gal concentration of 6.52 mM.

The global optimum of the specific activity is shown in Figure 5 in form of three contour plots illustrating the influence of temperature and pH at three different enzyme loadings of the carriers.

As can be expected the specific activity increases with increasing loading as well as increasing temperature. The slope of the pH influence is only moderate and shows an optimum pH value about 7, which is about 0.5 lower as could be expected from the first rough screening shown in Figure 4A. Besides the presented model considering linear and quadratic effects of the parameters, an extended model considering also interactions terms of the parameters has been fitted to the data. However, the fit didn't improve and the reported summary indicated a reduced quality of the model (data not shown). An indication of the independence of the influence of the investigated parameters can also be seen in the fact, that the overall appearance of the three contour plots is basically the same and only the absolute numbers are shifted by increasing the enzyme loading of the magnetic beads.

### Validation of Integrated Online Analytics

In order to validate the photometric online analytics integrated in the modular microreactor device, the specific activity of immobilized β4GalT at different temperatures was measured by using a continuous photometric assay according to Laaf et al. (2017), with the integrated spectrometer module. As a control, the same samples were measured by HPLC. The activities obtained this way (Table 2), had an average relative standard deviation of 4.6%, with the highest deviation of 7.5% at 30°C and the lowest of 2.3% at 35°C. With this result, the accuracy of the online analytic of photometric enzyme reactions in the reactor system was displayed.

**Table 2:** Comparison of the specific activity of immobilized β4GalT at different temperatures, measured by decrease of NADH+H^+^ according to Laaf et al. (2017) and standard measurement of the product by HPLC.

**Temperature**		**30°C**	**35°C**	**40°C**
Spec. activity [U/mg]	Reactor spectrometer	0.50	0.95	1.75
Measured with	HPLC	0.56	0.92	1.65
Relative standard deviation		7.53%	2.30%	3.90%

**Figure 6:**
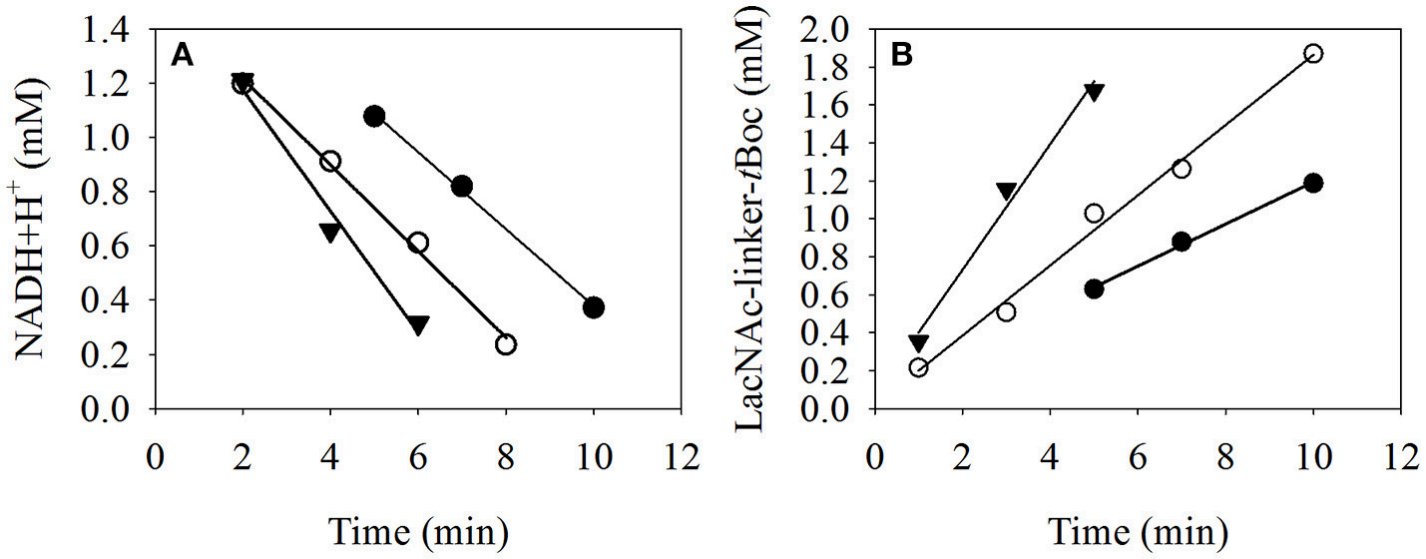
Kinetic analysis of the LacNAc-linker-*t*Boc formation with immobilized β4GalT **(A)** using the online photometer of the microreactor device to measure the decrease of NADH+H^+^ concentration as an effect of the side reactions described by Laaf et al. (2017) and **(B)** with the HPLC at 30°C (-•-), 35°C (-◦-), and 40°C (-▾-). At each sample point, the MEI were separated by a permanent magnet array and the supernatant was transferred to the UV/VIS module. Subsequently to the measurement at 340 nm, the supernatant was pumped back to the position of the MEI, which were resuspended in the solution by an alternating electromagnetic field. As a control, the samples were also measured with HPLC at 254 nm to analyze the product LacNAc-linker-*t*Boc.

To determine the process kinetics of immobilized β4GalT with the spectrometer module (Figure 6), the reaction solution of the reaction compartment can be separated virtually without loss from the MEI particles, measure NADH+H^+^ concentration in the solution and reunite it with the particles. This is a clear advantage compared to e.g., liquid handling stations, where particle separation and subsequent analysis of the remaining solution always is associated with unavoidable substance losses in pipetting tips and additional micro well plates.

### Demonstration of Multi-Cycle Reuse of Magnetic Enzyme Immobilisates

**Figure 7:**
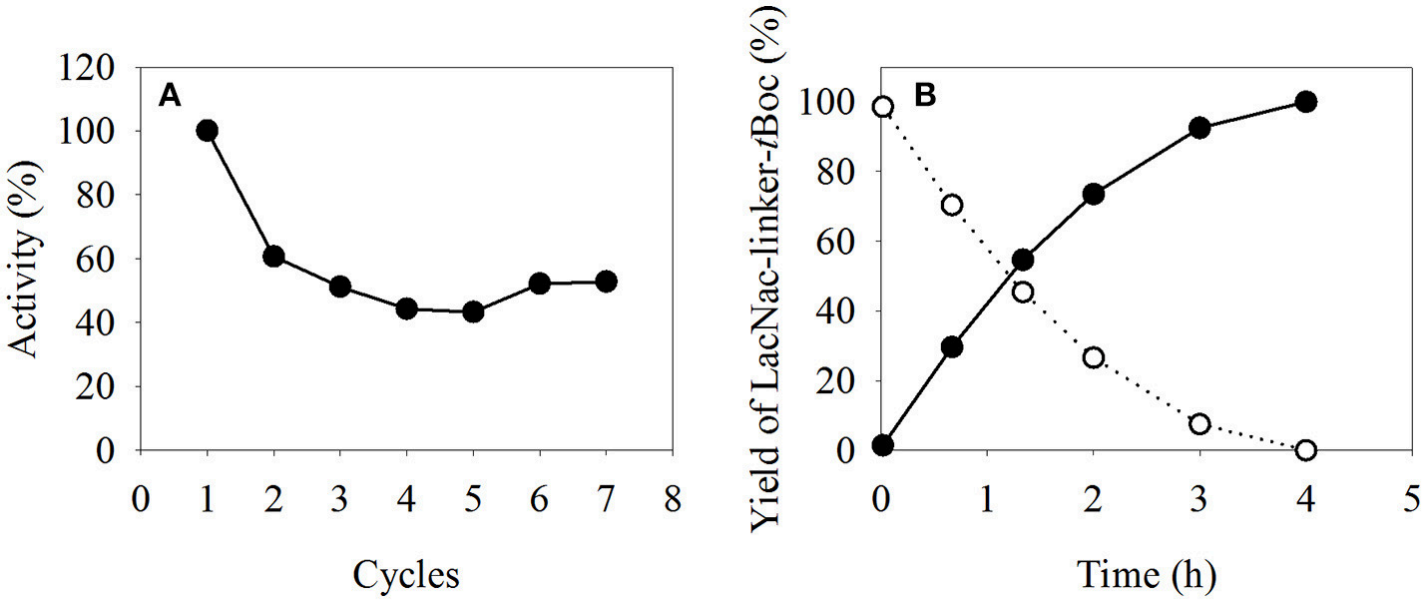
**(A)** Reuse of immobilized β4GalT in multiple cycles and **(B)** a full conversion of GlcNAc-linker-*t*Boc (-◦-) to LacNAc-linker-*t*Boc (-•-). The immobilisates were assayed seven times, with a washing step after each reaction. The assays were performed with 15 g/L MEI at 30°C with 100/25 mM HEPES/KCl (pH 7.5) and **(A)** 6.52 mM MnCl_2_, 6.52 mM UDP-Gal, 5 mM GlcNAc-linker-*t*Boc, and 1 U Fast-AP; **(B)** 15 mM MnCl_2_, 15 mM UDP-Gal, 10 mM GlcNAc-linker-*t*Boc, and 2 U Fast-AP.

The reusability of β4GalT immobilized on magnetic particles was determined in the microreactor by washing the assayed MEI with reaction buffer after each cycle and contacting them with fresh substrate solution. In this manner, the activity of immobilized β4GalT was analyzed seven times (Figure 7A). Setting the first measured activity as 100%, the activity dropped to 61% in the second cycle. However, in the following five assays, the activity remained around 50%. Combining the results of a full conversion to LacNAc-linker-*t*Boc (Figure 7B) with the multi-cycles results, a productivity of 0.84 g^*^L^−1*^h^−1^ was calculated. Estimating an 8 h working day using a 3 mL compartment, 20 mg of product per reaction tube of the TCM can be synthesized. In combination with the higher thermal stability (see Figure 4B), the recycling of MEI offer the possibility of automated, parallelized and more economic biocatalytic reactions in preparative lab scale.

## Conclusions

A compact, modular, and scalable microreactor device has been presented. The device uses magnetic carriers in compartmented aqueous solutions, which are contactless controlled by syringe pumps in combination with permanent and electromagnetic fields. As a proof of concept for the use as an automated device to optimize reaction conditions of enzymes, recombinant human β1,4-galactosyltransferase-1 was immobilized on magnetic microparticles and analyzed its performance/activity at different temperatures, pH values, substrate concentrations, and enzyme loadings. We demonstrate the accuracy of the developed temperature control, the reproducibility of the aqueous compartments, the efficiency of the integrated online analytics, the successful optimization of the reaction parameters with the reactor device and standard deviations of 0,02–0,3 U/mg, and the reusability of the immobilisates. The ability of frequent separation and reunification of the enzyme carriers and the reaction compartments without losses allows online monitoring of the reaction progress with photometric methods and the automated conduction of enzymatic cascades. Possible application fields of this reactor device could be biomedicine, biopharmacy, and food industry. As presented here, glycans could be automatically synthesized with this device in small quantities to be used e.g., for drug screening or as analytical standards of human milk oligosaccharides. In comparison to conventional liquid handling stations, the developed system also provides the advantage of a fully closed system avoiding solvent evaporation, the risk of contamination involved when handling e.g., open 96 well plates, and the danger resulting from harmful substrates, e.g., cyanides, used in biocatalytic reactions with immobilized enzymes.

## Author Contributions

RH and MF conceived and planned the experiments. JH and MF designed the original microreactor system. LE and TF provided the enzyme and the fundamental enzyme assays. RH carried out the experiments. RH and MF wrote the manuscript with input from all authors. All authors provided critical feedback and helped shape the research, analysis and manuscript.

### Conflict of Interest Statement

The authors declare that the research was conducted in the absence of any commercial or financial relationships that could be construed as a potential conflict of interest.
